# Effect of Annoying Sounds on Postural Control

**DOI:** 10.3390/jcm13092638

**Published:** 2024-04-30

**Authors:** Michalina Błażkiewicz, Michalina Gulatowska, Anna Hadamus, Justyna Kędziorek, Grażyna Brzuszkiewicz-Kuźmicka

**Affiliations:** 1 Faculty of Rehabilitation, The Józef Piłsudski University of Physical Education in Warsaw, 00-968 Warsaw, Poland; michalina.blazkiewicz@awf.edu.pl (M.B.);; 2 Department of Physiotherapy Fundamentals, Faculty of Dental Medicine, Medical University of Warsaw, 02-091 Warsaw, Poland

**Keywords:** noise annoyance, sound, postural control, nonlinear measures, CoP, body balance, imbalance

## Abstract

**Objectives**: This study aimed to explore the impact of irritating sounds on the postural control of healthy adults, considering both linear and nonlinear parameters, subjective assessments, and gender differences. **Methods:** Thirty-four young participants (17 females, 17 males) completed three 30 s bipedal standing stability tests on a balance platform: one with visual control (EO), another without visual control (EC), and a third without visual control but accompanied by irritating sounds (ECS). Additionally, participants filled out a questionnaire evaluating their sound sensitivity. Linear and nonlinear parameters from each balance test were considered for statistical analysis. **Results:** The findings reveal significant gender-based variations in sensitivity to sound, with women exhibiting higher sensitivity. No statistically significant differences in postural control were observed between males and females, except for a notable increase in irregularity (SampEn values) in the anterior–posterior direction for females in the ECS trial. Correlation analyses revealed a moderate and statistically significant correlation between SampEn values in the AP direction and SE scores. **Conclusions:** This study highlights the intricate relationship between sensory stimuli, attention, and the body’s ability to maintain balance. The presence of irritating sounds led to increased irregularity in postural control, particularly in the absence of visual control.

## 1. Introduction

Postural control is a multisensory system that relies on the synergism of visual, vestibular, and sensorimotor systems [1]. Each individual employs various somatosensory strategies to manage postural control, causing the dominance of visual, auditory, proprioceptive, and vestibular inputs to differ among people during their growth [1]. However, it is worth noting that due to the close proximity of the phonoreceptors and the vestibular organ, few studies [1,2,3] have shown that sound can affect postural control in adults. A sound is a wave created by vibrations that propagate as an acoustic wave through a transmission medium such as gas, liquid, or solid. In human physiology and psychology, sound is the reception of such waves and their perception by the brain [4]. The ear is adapted to discriminate between different characteristics of sound, such as pitch and loudness, which correspond to the frequency of sound waves and the perception of sound intensity, respectively [5]. The human ear can detect frequencies from 1000 to 4000 Hz. The sound intensity for the human ear ranges from 0 to 130 dB (where sound becomes painful). All these physical properties must undergo a transformation to reach the central nervous system [5].

Abrupt or sudden sounds have been demonstrated to induce postural destabilization [2,3,6,7]. Park et al. [7] showed that the magnitudes of postural body sway increased with increasing sound frequency, with higher disturbances around 4000 Hz. At frequencies of around 2000 Hz, the length of the postural sway was at a minimum variability. Moreover, it has been shown that sway area decreases for trials with high (1000 Hz) and very high (4000 Hz) frequencies [2]. Alessandrini et al. [8] showed that sounds of intensities below 90 dB had no effect on postural control. On the other hand, Halmagyi et al. [4] demonstrated that a constant tone above 95 dB can produce a postural deviation towards the stimulated ear. Agaeva and Altman [6] showed that the duration of a sound influences postural control. Also, increased exposure increases postural sway [7]. The impact of sound was critical when the exposure lasted at least 30 s [3,8,9]. No responses were observed when the sound stimulus was heard for less than 20 s [8].

It is worth noting that previous studies on the effect of sound on postural stability have not dealt with the influence of sound type (pleasant, irritating) on postural stability and also have not applied postural stability analysis using nonlinear measures, which, as has been repeatedly proven [10], are a more sensitive tool that can capture changes that are not apparent with linear measures. Given that irritating sounds refer to auditory stimuli that are bothersome, disruptive, or unpleasant to an individual, it is crucial to understand how they might affect postural stability. These sounds can act as distractors, leading to changes in sensory processing and attentional focus, which may impact postural stability mechanisms. Further research into these interactions may provide deeper insights into how auditory stimuli affect postural stability. The nonlinear measures, primarily sample entropy (SampEn), fractal dimension (FD), and Lyapunov exponent (LyE), play a crucial role in assessing various aspects of human behavior and physiological responses [10]. SampEn assesses the predictability or regularity of patterns present in the data. Specifically in relation to attention, SampEn can indicate the level of variability or disorder in physiological signals associated with cognitive processes. In tasks requiring attention, changes in sample entropy can reflect fluctuations in a person’s attentional state. Higher SampEn may suggest greater cognitive engagement or a more complex processing mode. FD is a mathematical concept used to describe an irregular, self-similar, or complex data structure. The FD can assess the complexity of postural sway patterns. A higher FD in the analysis of postural control may indicate a more adaptive and flexible control strategy to accommodate different environmental or task-related challenges. The LyE is a measure that quantifies the rate of divergence or convergence of trajectories in a dynamic system. Regarding body motion and stability, the LyE can offer insights into the rigidity or stability of the body’s dynamics. A lower LyE might suggest more stable and predictable movements, while higher values could indicate greater variability or less predictable motion in the body’s dynamics. These nonlinear measures provide valuable insights into different aspects of human performance and physiological responses, offering a more nuanced understanding beyond traditional linear measures. They help capture complexities, variability, and subtleties in behavior and physiological processes, contributing to a deeper comprehension of attention, postural control, and bodily dynamics. Therefore, this study aimed to investigate how irritating sounds affect the balance of healthy adults.

## 2. Materials and Methods

### 2.1. Participants and Measurement Protocol

Thirty-four subjects (17 females and 17 males) were enrolled in this study (Table 1). Inclusion criteria for this study were as follows: no usage of medications affecting stability and the capability to maintain balance, as well as the absence of any balance system or auditory system-related diseases. All participants gave their informed consent to participate in the research, which had previously been approved by the university’s institutional review board (No. SKE01-15/2023). This study followed ethical guidelines and the principles of the Declaration of Helsinki.

**Figure 1:**
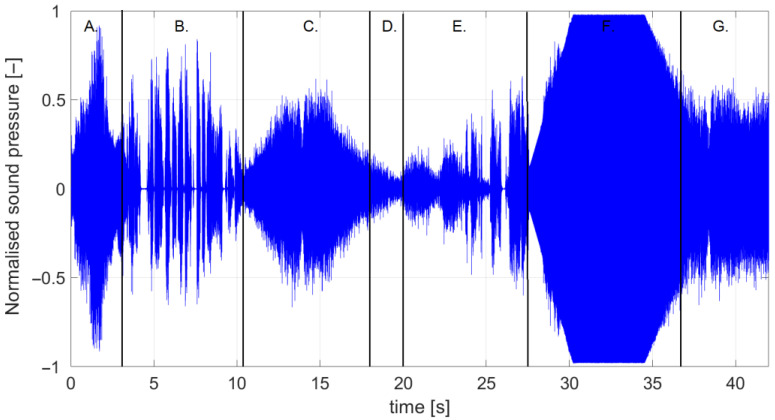
The appearance of the audible sound signal perceived by the subjects: A. vuvuzela, B. fork, C. chainsaw, D. ambulance, E. styrofoam, F. screech, and G. grinder.

The subjects underwent three stability assessment tests, which included bipedal standing with visual control both on and off (EO, EC), as well as with visual control off and a sequence of annoying sounds on (ECS). The soundtrack the subjects listened to consisted of sequential stereo sounds: vuvuzela, fork, chainsaw, ambulance, styrofoam, screech, and grinder (Figure 1). The sampling rate of the sound was 44.1 kHz, which means 44,100 samples per second.

Each of the three measurements lasted 30 s. During each measurement, subjects wore headphones over their ears to establish a standardized auditory environment, minimizing external distractions that could potentially influence the experimental outcomes. During the test with visual control, subjects looked at a fixed point at eye level on a wall located 1.5 m away. An additional trial without visual support was conducted to ensure that any observed effects would be attributed solely to auditory rather than visual stimuli (focusing on a point). Moreover, during each trial, the subject stood on the platform in the same place in their foot outlines so that the base of support was always the same within one subject. The feet were positioned at hip width, and the outline of the feet was marked on the platform before the first measurement. Before each measurement, a calibration procedure was conducted following the manufacturer’s guidelines [11]. Calibration was performed with the subject off the platform. It commenced with the elimination of any offsets. Next, the designated area on the platform, marked previously to indicate where the test person would stand, was identified. The subsequent step involved taking measurements. The postural stability data for each subject were recorded using an AMTI AccuSway (Advanced Mechanical Technology Inc., Watertown, MA, USA) plate with Balance Clinic 2.02.01 software. The sample rate was set at 100 Hz, as recommended by Stergiou [12]. This frequency is best for papers where nonlinear measures are calculated [10]. Moreover, data files were saved in their raw, unfiltered state.

### 2.2. Hearing Hyperacusis Questionnaire (HQ)

**Figure A1:**
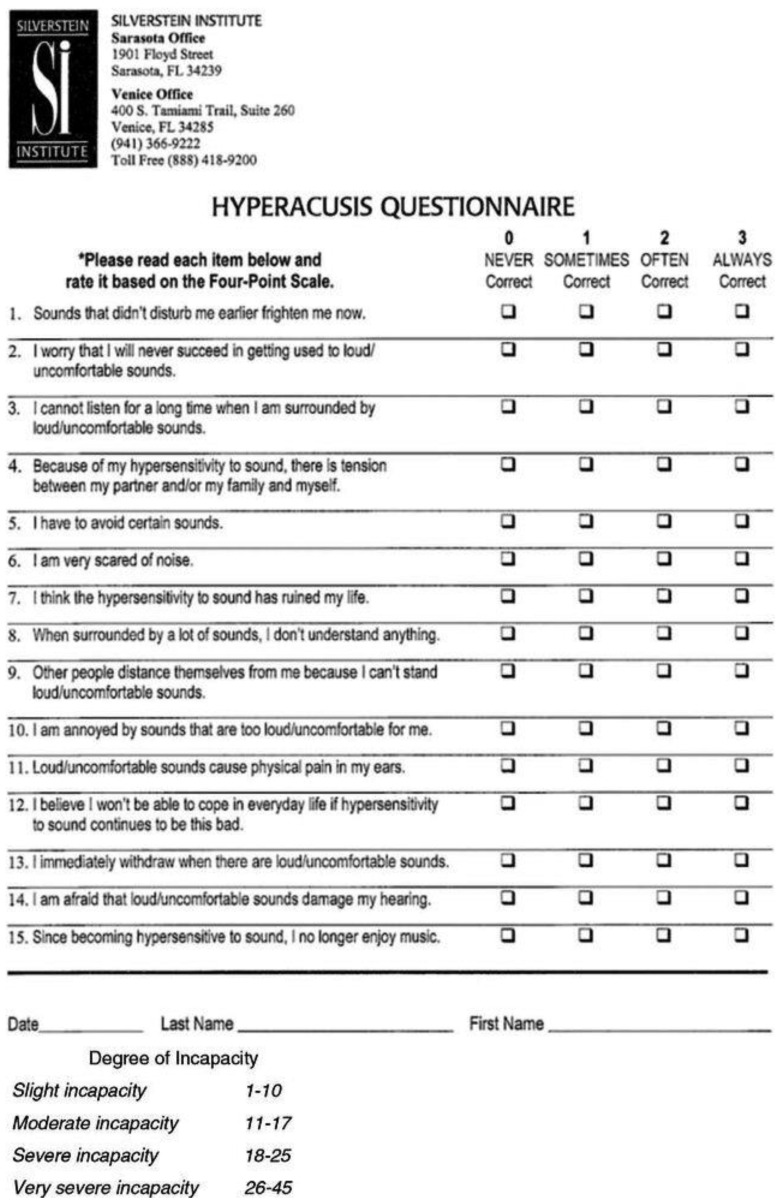
The hyperacusis questionnaire [13,20].

Subjects were asked to complete a questionnaire assessing their sensitivity to sound before participating in this study [13,14]. This questionnaire comprises 15 items, assessing three specific dimensions or subscales: cognitive behavior associated with hyperacusis, somatic reactions linked to particular situations, and emotional responses [13]. Respondents use a four-point Likert-type scale, answering ‘no’ (0 points), ‘yes, a little’ (1 point), ‘yes, quite a lot’ (2 points), and ‘yes, a lot’ (3 points) for each item. The total score range possible is from 0 to 45, with higher scores indicating a greater degree of sound hypersensitivity. The complete questionnaire is in Appendix A, Figure A1.

In addition, on a scale of 1–10, participants rated the degree of annoyance of the sound turned on during the test, where 1 meant no annoyance and 10 meant very disturbing and annoying.

### 2.3. Assessed Linear and Nonlinear Parameters

From each measurement, a set of linear and nonlinear parameters were considered. Among the linear parameters, the following were included: the center of foot pressure path length (CoP), the CoP path length in the anterior–posterior (AP) and mediolateral (ML) directions, and velocities.

The nonlinear parameters analyzed were: sample entropy (SampEn), fractal dimension (FD), and Lyapunov exponent (LyE). All coefficients were calculated using MatLab software v. R2021a (MathWorks, Natick, MA, USA) for 30 s samples covering 3000 points in each direction.

SampEn is the negative natural logarithm of the conditional probability that a dataset of length *N*, having repeated itself within a tolerance *r* for *m* points, will also repeat itself for *m* + 1 points, without allowing self-matches:

$$SampEn\left(m,r,N\right)=-ln\left(\frac{A^{m}\left(r\right)}{B^{m}\left(r\right)}\right),$$

where *B* represents the total number of matches of length *m* while *A* is the subset of *B* that also matches for *m* + 1. For calculating the *SampEn*, MatLab codes obtained from the Physionet tool [15] were used, with “default” parameter values: *m* = 2 and *r* = 0.2 × (standard deviation of the data).

FD was calculated using Higuchi’s algorithm [16]. Higher FD values are associated with the greater complexity of a time series.

LyE was calculated to detect chaotic system dynamics using the following equation:

$$d\left(t\right)={Ce}^{LyEt}$$

where *d*(*t*) is the average divergence at time *t* and *C* is a constant that normalizes the initial separation [17]. A positive Lyapunov exponent (LyE) value is an essential indication or requirement for the existence of chaos within a specific system. If LyE is zero, the system is conservative (i.e., no dissipation). If the system is dissipative, the LyE value is negative.

### 2.4. Statistical Analysis

Statistical analysis was conducted using PQStat 2021 software v. 1.8.2.238 (PQStat Software, Poznań, Poland). The threshold for statistical significance was set at *p* < 0.05. The Shapiro–Wilk test was used to assess the normality of all data distributions. The U Mann Whitney test was employed to determine statistically significant differences between male (M) and female (W) outcomes for both postural stability, subjective ratings of the sound annoyance (SE) and questionnaire scores assessing sensitivity to sound (HQ).

An ANOVA Friedman with Dunn Bonferroni post hoc test was performed to examine the effects of disabling visual control and both disabling visual control and annoying sounds on postural control.

Spearman correlation coefficients (r) were calculated separately for men and women, as well as for the entire group, to assess the relationship between the values of linear and nonlinear parameters, the scores from the HQ test, and the subjective ratings of the sound annoyance experienced during the test. Spearman’s correlation coefficient is a statistical measure of the strength of a monotonic relationship between paired data and is in the range −1 ≤ r ≤ 1 (Table 2) [18].

## 3. Results

After the Shapiro–Wilk test was performed, it was shown that most of the parameters had a distribution different from normal. This imposed the need to use non-parametric tests in further analysis.

### 3.1. Gender Differences

After the U Mann Whitney test was conducted, no statistically significant differences were found between the linear and nonlinear parameter values during the standing test with eyes open and closed. However, within the trial involving closed eyes and disturbing sounds (Table 3), SampEn values in the AP direction were significantly higher in the female group compared to those noted in the male group (0.08 ± 0.03 vs. 0.06 ± 0.02, *p* = 0.01).

Taking into account the subjective evaluation of the test participants, it was shown that women rated the sound significantly more annoying than men (8.47 ± 1.37 vs. 6.59 ± 1.84, *p* < 0.001), which is in line with the results of the Hearing Hypersensitivity Questionnaire (HQ). The outcomes from the HQ indicated that the study group exhibited scores of 9.85 ± 7.73, falling within the category of slight incapacity. Among the group, the highest score was achieved by a woman, totaling 32 points, indicating a classification of very severe incapacity. In contrast, the lowest score, 2 points, was recorded by a man and categorized as slight incapacity. In summary, scores from the hyperacusis questionnaire were significantly higher in the female group than in the male group (13 ± 8.7 vs. 6.71 ± 5.16, *p* = 0.03) (Table 3).

### 3.2. Effects of Disabling Visual Control and Both Disabling Visual Control and Annoying Sound on Postural Control

The conducted analysis revealed that across all linear and nonlinear parameters, the highest values were observed during the trial with visual control off and an annoying sound, and the lowest values were observed during the trial with eyes open. However, after ANOVA Friedman with Dunn Bonferroni post hoc analysis was conducted, statistically significant differences emerged primarily between trials involving visual control on and off and between trials with visual control on and visual control off and annoying sound (Table 4).

Among the nonlinear parameters, only SampEn in the anteroposterior (AP) direction exhibited statistically significant differences across all trials. However, it is also worth noting that among the nonlinear parameters, the main differences were noted for the AP direction. In the ML direction, the values of nonlinear parameters were at similar levels, excluding LyE, whose values for the ECS sample were significantly higher than those recorded for the EO sample (Table 4).

### 3.3. The Spearman Correlation Results

The Spearman correlation results revealed weak or very weak associations (Table 2 and Table 5) between the linear and nonlinear parameters and HQ and SE test scores.

Interestingly, moderate monotonic correlations were found between the CoP displacement velocity in both the AP and ML directions and the subjective ratings of sound annoyance in the male (r = 0.45) and female (r = 0.43) groups, respectively. Unfortunately, none of the correlations mentioned were statistically significant (*p* > 0.05).

Regarding nonlinear parameters, moderate monotonic correlations were observed between SampEn and LyE values in the AP direction and SE scores for the overall (r = 0.41) and the female (r = 0.46) groups, respectively. It is worth noting that the correlation between SampEn values in the AP direction and SE scores was the only statistically significant one.

In addition, consistently negative correlation values were observed for all nonlinear parameters. These correlations were not statistically significant and mostly fell into the very weak and weak categories. Their negative values suggest that as the values of the nonlinear parameters increased, there was a corresponding decrease in sound sensitivity across all groups and questionnaire measurements (Table 5).

## 4. Discussion

The aim of this study was to investigate how irritating sounds affect the postural control of healthy adults. Postural stability was evaluated across three tests, which encompassed bipedal standing scenarios with eyes open, eyes closed, and eyes closed in the presence of an irritating sound. Individuals were assessed for postural control using linear and nonlinear parameters calculated in the anterior–posterior (AP) and mediolateral (ML) directions across each test. Additionally, participants provided subjective ratings of sound annoyance on a scale from 1 to 10 [19] and completed the Hearing Hypersensitivity Questionnaire [13,20].

The results indicated a significant difference in sensitivity to sound between genders, with women displaying significantly higher sensitivity in both subjective ratings and Hearing Hypersensitivity Questionnaire scores. This finding aligns with the results obtained in the paper by Yilmaz et al. [21]. Also, Hasson et al. [22] and Baribeau [23] demonstrated that women tend to be more sensitive to sounds, experiencing heightened emotional or auditory distress due to hormonal variations or shifts in emotional states. Consequently, women might perceive sounds as more threatening and direct their attention toward the negative impacts of noise, potentially resulting in hyperacusis.

No statistically significant differences were found when assessing postural stability in the tests studied between the male and female groups. However, within the trial involving closed eyes and disturbing sounds, SampEn values in the AP direction were notably higher, showing a 33.33% increase compared to those recorded in the male group. This outcome indicates a greater irregularity of the CoP signal along this axis, which appears more random and less predictable, probably only due to disturbing sound. The dominance of swings in the sagittal plane was also shown in the paper of Park et al. [7], which discussed the effect of sound on postural control. Savard et al. [24] showed that sudden loud or unexpected irritating sounds might provoke more pronounced postural responses compared to continuous, low-level annoying noises. Overall, the effect of annoying sounds on postural control highlights the intricate relationship between sensory stimuli, attention, and the body’s ability to maintain balance.

When considering the entire group comprising both males and females, the analysis showed no statistically significant differences in the set of linear parameters calculated for trials of standing with eyes closed and standing without visual control while exposed to a disturbing sound. Notably, this trend persisted across most nonlinear parameters, except for the SampEn values in the AP direction. In the presence of the sound signal, these values exhibited a significant increase of 16.66% for the entire group. Clearly, closing the eyes led to a notable increase in all linear parameters compared to values observed during free-standing with visual control. This outcome aligns with the majority of findings in the field of postural stability assessment [10]. A similar pattern emerged when comparing the open-eyes free-standing test with the trial involving no visual control and the presence of a disturbing sound. However, this trend in nonlinear parameters was only consistent for the AP direction. In the ML direction, there were no statistically significant differences between the tests. Notably, only the values of the Lyapunov exponent in the ML direction during standing with visual control and disturbing noise exhibited a significant 5% increase compared to those recorded in the baseline trial (eyes open). This result indicates the potential for a quicker balance control response in various body movements, also in the ML direction, extending beyond the previously observed effects primarily seen in the AP direction.

Weaving into the area of correlation, no strong correlations emerged between stability scores measured by linear and nonlinear parameters and the questionnaire or subjective scores in the postural control assessment test in the trial without visual control and sound presence. Two moderate correlations emerged in the set of linear parameters. First, r = 0.45 was observed between the velocity of CoP motion in the AP direction and subjective ratings of sound annoyance within the male cohort. Similarly, a correlation (r = 0.43) of comparable strength appeared between the speed of CoP movement in the ML direction and subjective ratings of sound annoyance among the female participants. Nonetheless, these findings contrasted with the correlations derived from HQ scores, which exhibited weaker associations of 0.27 and 0.36, respectively. However, none of these correlations were statistically significant. The implication drawn from these results suggests that as the speed of movement in a specific direction increases, there is an apparent escalation in sensitivity to irritating sounds. Consequently, these outcomes imply a potential connection between the velocity of these movements and an individual’s inclination to perceive certain sounds as bothersome or irritating. However, the described pattern is not robust enough to meet the criteria for statistical significance.

Among the nonlinear parameters, moderate correlations were identified between SampEn and LyE values in the AP direction and subjective evaluation scores for the general (r = 0.41) and female (r = 0.46) groups, respectively. This observation implies that with increasing SampEn and LyE values, there is a heightened sensitivity to annoying sounds. However, the correlation between SampEn values toward AP and SE scores observed in the entire group was the only statistically significant one. This means that despite not being exceptionally strong, the correlation is robust enough to confidently conclude that the relationship observed in the data is unlikely to be due to random chance alone. Thus, in the general group, as the irregularity of the CoP signal waveform intensifies in the AP direction, there is an associated increase in sensitivity to irritating sounds. On the other hand, within the female group, there appears to be a more prompt reaction to increasingly intolerable sounds, as indicated by the LyE values. It is worth noting that in the group of nonlinear parameters, negative correlations were frequently observed. Despite falling into the categories of very weak and weak correlations, they suggest that as the values of nonlinear parameters increase, there is a corresponding decrease in sensitivity to sound across all groups and questionnaire measurements.

This study has several limitations, which also contribute value by showing the way for future research directions to complement the current approach. Firstly, the sample size was relatively small, which may limit the generalizability of reported findings to larger populations. Additionally, while efforts were employed to control for potential confounding variables, there may still be unaccounted factors that may have influenced results. Furthermore, the methodology employed in this study may have inherent biases or limitations that could affect the interpretation of the findings. Readers may discover further limitations beyond those mentioned here; however, these will continually serve as new directions and sources of inspiration to enhance and complete this study and its approach.

## 5. Conclusions

This research demonstrated that the impact of irritating sounds on postural control disrupts established stability by functioning as a distractor. These sounds induced alterations in sensory processing and attentional focus, subsequently influencing mechanisms governing postural stability. Additionally, individual responses to annoying sounds can vary, influenced by factors such as sound intensity, frequency, individual susceptibility, and the particular context in which the sounds are encountered. Further research into these interactions can provide deeper insights into how auditory stimuli impact postural stability and potentially inform strategies for mitigating the adverse effects of such sounds on posture and balance. Future investigations should consider factors such as sound characteristics (specification, intensity, frequency), along with how they relate to weight distribution and muscle activity.

## Figures and Tables

**Table 1:** Characteristics of the participants (mean ± standard deviation).

Group	Age [Years]	Body Weight [kg]	Body Height [cm]
Women (*n* = 17)	22.24 ± 3.01	63.12 ± 13.83	168.94 ± 6.71
Men (*n* = 17)	20.59 ± 1.33	74.65 ± 11.52	179.47 ± 8.47
All (*n* = 34)	21.41 ± 2.44	68.89 ± 13.83	174.21 ± 9.23

**Table 2:** The strength of Spearman’s correlation coefficient [18].

Classification Values	Interpretation (Measure of Strength)
r = 0	no correlation
0 < |r| < 0.20	very weak
0.20 ≤ |r| < 0.40	weak
0.40 ≤ |r| < 0.60	moderate
0.60 ≤ |r| < 0.80	strong
0.80 ≤ |r| < 1	very strong
r = 1	monotonic correlation

**Table 3:** Mean and standard deviation of linear and nonlinear parameters in standing trials with eyes closed and annoying sound (ECS), sound annoyance assessed by Hearing Hypersensitivity Questionnaire (HQ), and subjective ratings of the sound annoyance experienced during the test (SE). The last column contains *p*-values for the U Mann Whitney test indicating the presence of statistically significant differences.

Parameters	ECS W	ECS M	U Mann Whitney Test *p*-Value
CoP path length [mm]	352.12 ± 159.9	285.88 ± 105.42	-
CoP path length AP [mm]	277.59 ± 136.63	224.41 ± 92.49	-
CoP path length ML [mm]	161 ± 65.62	131.71 ± 48.71	-
CoP velocity [mm/s]	11.74 ± 5.32	9.54 ± 3.51	-
CoP velocity AP [mm/s]	9.7 ± 5.18	8.22 ± 3.46	-
CoP velocity ML [mm/s]	5.28 ± 3.03	4.68 ± 2.56	-
SampEn AP [-]	0.08 ± 0.03	0.06 ± 0.02	*p* = 0.01
SampEn ML [-]	0.08 ± 0.03	0.08 ± 0.03	-
FD AP [-]	1.25 ± 0.07	1.24 ± 0.07	-
FD ML [-]	1.28 ± 0.08	1.27 ± 0.06	-
LyE AP [-]	1.44 ± 0.19	1.38 ± 0.11	-
LyE ML [-]	1.14 ± 0.27	1.02 ± 0.21	-
HQ [point]	13 ± 8.7	6.71 ± 5.16	*p* = 0.03
SE [point]	8.47 ± 1.37	6.59 ± 1.84	*p* < 0.001
HQ ALL [point]	9.85 ± 7.73		-
SE ALL [point]	7.53 ± 1.86		-

CoP—center of pressure, AP—anterior–posterior direction, ML—mediolateral direction, SampEn—sample entropy, FD—fractal dimension, LyE—Lyapunov exponent, ECS—standing trials with eyes closed and annoying sound, HQ—Hearing Hypersensitivity Questionnaire, SE—subjective ratings of the sound annoyance, ALL—all participants, W—women, M—men.

**Table 4:** Mean and standard deviation of linear and nonlinear parameter values for standing trials with eyes open (EO), eyes closed (EC), and eyes closed and annoying sound (ECS). The last three columns contain *p*-values for the Dunn Bonferroni post hoc test indicating the presence of statistically significant differences.

Parameters	EO	EC	ECS	EO vs. EC	EO vs. ECS	EC vs. ECS
Linear						
CoP path length [mm]	247.03 ± 65.79	298.68 ± 89.34	319 ± 137.54	*p* < 0.001	*p* < 0.001	-
CoP path length AP [mm]	185.15 ± 47.5	231.35 ± 67.08	251 ± 118.01	*p* < 0.001	*p* < 0.001	-
CoP path length ML [mm]	125.18 ± 42.64	141.15 ± 53.25	146.35 ± 58.92	*p* = 0.03	*p* = 0.02	-
CoP velocity [mm/s]	8.24 ± 2.19	9.96 ± 2.97	10.64 ± 4.58	*p* < 0.001	*p* < 0.001	-
CoP velocity AP [mm/s]	6.15 ± 1.93	8.47 ± 2.71	8.96 ± 4.41	*p* < 0.001	*p* < 0.001	-
CoP velocity ML [mm/s]	4 ± 1.87	4.82 ± 2.17	4.98 ± 2.78	-	-	-
Nonlinear						
SampEn AP [-]	0.05 ± 0.02	0.06 ± 0.02	0.07 ± 0.02	*p* = 0.04	*p* < 0.001	*p* = 0.01
SampEn ML [-]	0.08 ± 0.03	0.08 ± 0.03	0.08 ± 0.03	-	-	-
FD AP [-]	1.21 ± 0.08	1.24 ± 0.07	1.25 ± 0.07	*p* = 0.02	*p* = 0.005	-
FD ML [-]	1.26 ± 0.07	1.27 ± 0.06	1.28 ± 0.07	-	-	-
LyE AP [-]	1.32 ± 0.13	1.41 ± 0.15	1.41 ± 0.15	*p* = 0.007	*p* = 0.005	-
LyE ML [-]	1.03 ± 0.21	1.06 ± 0.25	1.08 ± 0.24	-	*p* = 0.003	-

CoP—center of pressure, AP—anterior–posterior direction, ML—mediolateral direction, SampEn—sample entropy, FD—fractal dimension, LyE—Lyapunov exponent, EO—standing trials with eyes open, EC—standing trials with eyes closed, ECS—standing trials with eyes closed and annoying sound.

**Table 5:** The Spearman’s rank correlation coefficients (r) and *p*-value between parameters assessing stability and sound disturbance scores provided by the Auditory Hypersensitivity Questionnaire (HQ) and subjective ratings of sound annoyance (SE).

Parameters	r ECS ALL	r ECS W	r ECS M
CoP path length [mm]	HQ: 0.21, *p* = 0.22 SE: 0.29, *p* = 0.09	HQ: 0.27, *p* = 0.28 SE: 0.35, *p* = 0.15	HQ: 0.01, *p* = 0.95 SE: 0.19, *p* = 0.46
CoP path length AP [mm]	HQ: 0.28, *p* = 0.10 SE: 0.33, *p* = 0.05	HQ: 0.31, *p* = 0.21 SE: 0.34, *p* = 0.17	HQ: 0.15, *p* = 0.54 SE: 0.26, *p* = 0.31
CoP path length ML [mm]	HQ: 0.17, *p* = 0.32 SE: 0.27, *p* = 0.12	HQ: 0.12, *p* = 0.62 SE: 0.27, *p* = 0.28	HQ: 0.04, *p* = 0.86 SE: 0.14, *p* = 0.57
CoP velocity [mm/s]	HQ: 0.21, *p* = 0.22 SE: 0.28, *p* = 0.09	HQ: 0.27, *p* = 0.29 SE: 0.35, *p* = 0.16	HQ: 0.03, *p* = 0.89 SE: 0.19, *p* = 0.46
CoP velocity AP [mm/s]	HQ: 0.27, *p* = 0.18 SE: 0.33, *p* = 0.06	HQ: 0.26, *p* = 0.31 SE: 0.29, *p* = 0.25	HQ: 0.27, *p* = 0.29 SE: 0.45 *, *p* = 0.06
CoP velocity ML [mm/s]	HQ: 0.23, *p* = 0.12 SE: 0.24, *p* = 0.17	HQ: 0.36, *p* = 0.14 SE: 0.43 *, *p* = 0.08	HQ: 0.07, *p* = 0.78 SE: 0.04, *p* = 0.85
SampEn AP [-]	HQ: 0.23, *p* = 0.19 SE: 0.41 *, *p* = 0.01 ^	HQ: −0.08 **, *p* = 0.74 SE: −0.04 **, *p* = 0.85	HQ: 0.22, *p* = 0.39 SE: 0.39, *p* = 0.11
SampEn ML [-]	HQ: −0.13 **, *p* = 0.44 SE: 0.01, *p* = 0.94	HQ: 0, *p* = 1 SE: −0.03, *p* = 0.90	HQ: −0.39 **, *p* = 0.12 SE: −0.18, *p* = 0.48
FD AP [-]	HQ: −0.07 **, *p* = 0.67 SE: 0.13, *p* = 0.46	HQ: −0.03 **, *p* = 0.88 SE: 0.12, *p* = 0.62	HQ: −0.19 **, *p* = 0.45 SE: 0.11, *p* = 0.66
FD ML [-]	HQ: −0.29 **, *p* = 0.09 SE: −0.08 **, *p* = 0.65	HQ: −0.27 **, *p* = 0.29 SE: −0.06 **, *p* = 0.81	HQ: −0.38 **, *p* = 0.12 SE: −0.16 **, *p* = 0.52
LyE AP [-]	HQ: 0.21, *p* = 0.22 SE: 0.19, *p* = 0.28	HQ: 0.39, *p* = 0.12 SE: 0.46 *, *p* = 0.06	HQ: 0, *p* = 0.97 SE: −0.15 **, *p* = 0.56
LyE ML [-]	HQ: 0.13, *p* = 0.44 SE: 0.18, *p* = 0.30	HQ: 0.06, *p* = 0.81 SE: 0.22, *p* = 0.38	HQ: 0.08, *p* = 0.76 SE: −0.03 **, *p* = 0.89

CoP—center of pressure, AP—anterior—posterior direction, ML—mediolateral direction, SampEn—sample entropy, FD—fractal dimension, LyE—Lyapunov exponent, ECS—standing trials with eyes closed and annoying sound, HQ—Hearing Hypersensitivity Questionnaire, SE—subjective ratings of the sound annoyance, ALL—all participants, W—women, M—men, r—Spearman’s rank correlation coefficients, * moderate association between variables, ** negative correlation values, ^ statistical differences.

## Data Availability

The measurement data used to support the findings of this study are available from the corresponding author upon request.
